# High Charlson Comorbidity Index Score is associated with early fracture-related complication for internal fixation of neck of femur fractures

**DOI:** 10.1038/s41598-022-08855-0

**Published:** 2022-03-19

**Authors:** Ronald Man Yeung Wong, Yao Zu, Wai Wang Chau, Chi Yin Tso, Wing Hong Liu, Raymond Wai Kit Ng, Simon Kwoon Ho Chow, Wing Hoi Cheung, Ning Tang, Kevin Ki Wai Ho

**Affiliations:** 1 Department of Orthopaedics and Traumatology, The Chinese University of Hong Kong, Hong Kong, China; 2 Department of Orthopaedics and Traumatology, Prince of Wales Hospital, Hospital Authority, Hong Kong, China

**Keywords:** Osteoarthritis, Prognosis

## Abstract

The incidence of geriatric hip fractures continues to rise in our aging population and has become a major public health concern globally. The primary outcome of this study was to determine whether Age-adjusted Charlson Comorbidity Index (ACCI) is associated with increased fracture-related complications in neck of femur fractures treated by internal fixation. This was a cohort study between January 2014 to June 2018. All patients ≥ 50 years old with an acute neck of femur fracture after low-energy trauma fixed with cannulated hip screws were included and followed-up for 1 year at a tertiary centre. Primary outcome was to determine whether ACCI was associated with increased fracture-related complications. Secondary outcomes were revision rate, mortality, and function after surgery. Further analysis were performed within a “younger” group (age 50–65) and “elder” group (age > 65), as displaced fractures (Garden Type III/IV) were in “younger” group. 233 hip fractures (68 males; 165 females) with a mean age of 73.04 ± 12.89 were included in the study. Surgical outcomes showed that the complication rate of hip screw fixation for all patients was 21.5% (50 patients) at 1 year. ACCI was significantly higher in all patients with complications (p = 0.000). Analysis within “younger” (p = 0.000) and “elder” groups (p = 0.006) both showed significance. Stepwise logistic regression modelling showed ACCI had positive correlation with complications with ACCI = 6 (OR 4.27, p = 0.02). R^2^ values were comparatively better after controlled by Garden Type III/IV at ACCI = 4 (OR 6.42 (1.70, 24.25), p = 0.01). The authors recommend that for patients with a Garden Type I/II and ACCI ≥ 6 or a Garden Type III/IV and ACCI ≥ 4, a direct arthroplasty surgery should be considered.

## Introduction

The incidence of geriatric hip fractures continues to rise in our aging population and has become a major public health concern globally. In the year 2000, there were 1.6 million hip fractures worldwide^1^ and it is projected by 2050 the number would rapidly increase and the majority will occur in Asia^2^. More importantly, a hip fracture is now the top 10 disability for adults, leading to a huge socioeconomic burden^3^. Mortality is also high with numbers reaching 30% in 1-year^4^. In China, the total costs of hospitalization was USD 380 million in 2016 with per patient average cost at USD 6840^4^. The mainstay of neck of femur hip fracture management is surgical fixation or arthroplasty for pain relief and early mobilization. Hip screws fixation is minimally invasive, time-saving and easy to perform^5^. It has well-known in literature that it has low intra-operative bleeding, and peri-operative complications, as compared to arthroplasties^6^. However, the need for re-operation for surgical fixation remains high in neck of femur fractures, ranging from 10–48.8%, warranting more evidence in the decision making for these patients^7^.

A major consideration is the age of the patient and displacement of the fracture, as arthroplasty is the preferred treatment of choice for a displaced fracture in patients 65 years or older due to a significantly lower risk of re-operation compared with internal fixation^8^. It is well known that in a displaced fracture, the blood supply to the femoral head is disrupted leading to a high risk of avascular necrosis and fracture non-union. On the other hand, in a non-displaced fracture, internal fixation is performed. In a “younger” patient, the treatment is with internal fixation in both displaced and non-displaced neck of femur fractures as the survivorship of an arthroplasty will unlikely last beyond 20 years^8,9^. However, there has been numerous studies with varying cut-offs for “younger” hip fractures mainly at 50^10,11^, 60^12,13^ and 65^9,14^ years old. Due to this, there has been reports of results in different age groups. Hong Kong has one of the longest life expectancies in the world^15^, and the general consensus for cut-off is at 65 years old^16^.

The optimal management of femoral neck fractures remain controversial in the age group between 50 to 65^8^. However, in a patient with multiple comorbidities, whether we should proceed to a direct arthroplasty procedure is also debatable. As a result, current guidelines in the best management of femoral neck fractures in these patients are still under study^8^. The Age-adjusted Charlson Comorbidity Index (ACCI) has mainly been used to predict mortality in hip fracture patients but is also strongly associated with frailty. It is also well known that these patients have poor bone quality and often osteoporosis^17^. Hip fractures are potentially life-changing and is the most serious form of osteoporotic fractures associated with a huge burden to our society^18^. With the increasing prevalence of osteoporosis, studies to update guidelines for hip fracture patients in the age of 50–65 are warranted. The primary outcome of this study was to determine whether ACCI is associated with increased fracture-related complications of neck of femur fractures treated by internal fixation.

## Methodology

This was a cohort study between January 2014 to June 2018. At our tertiary unit, Prince of Wales Hospital, The Chinese University of Hong Kong, data of all our operation records are recorded into the Clinical Management System database prospectively. This study was approved by The Joint Chinese University of Hong Kong—New Territories East Cluster Clinical Research Ethics Committee (CREC Ref. No. 2019.186). The study protocol is in compliance to Declaration of Helsinki and ICH-GCP. Informed consent was obtained from all subjects and/or their legal guardian(s). Similar to international guidelines, our standard practise is to perform surgery for neck of femur hip fractures patients within 48 h of admission unless medically unfit^8,19^. Internal fixation is used for “younger patients” and undisplaced fractures in older patients 65 years or older^8,19^. An ortho-geriatrician specialist identifies and treats correctable comorbidities and optimizes the medical condition for all our hip fracture patients. The inclusion criteria in this study were (1) patients 50 years or older, (2) suffered from a neck of femur fracture, (3) low-energy trauma, (4) internal fixation with cannulated hip screws. Exclusion criteria were (1) patients aged < 50 years old, (2) less than 1 year follow-up, (3) previous fracture on same femur, (4) open fracture, (5) pathological fracture. All recruited patients were subject to internal fixation with 3 cannulated hip screws in an inverted triangle fashion under fluoroscopy with an acceptable Garden alignment index^20^. Closed reduction was performed for all our patients. Follow-up for patients were 1 year. Information including patient demographics, functional status, fracture type (Garden Classification Type I/II, and Type III/IV)^21^, Age-adjusted Charlson Comorbidity Index (ACCI) and clinical outcomes were collected for analysis. The “younger” group was defined as age 50–65, and the “elder” group was defined as age above 65. The primary outcome of this study was to determine whether ACCI is associated with increased fracture-related complication rate of neck of femur fractures treated by internal fixation. A complication was defined as fracture non-union, avascular necrosis, screw cut-out, infection and periprosthetic fracture. The secondary outcomes were revision rate, mortality rate, and function after surgery.

### Sample size calculation

With reference to a cohort study using Charlson Comorbidity Index for hip fractures in elderlies^22^, the change in percentages with adjusted standard deviation trying to increase the data stringency. With an effect size of 0.17 reflecting the change in complication rate of 25% with halved standard deviations. Assuming a 10% drop-out, the required sample size to achieve an 80% power (β = 0.2) at α = 0.05 for correctly detecting such difference is 230 in total. The calculation was carried out using G*Power 3.1.9.

### Bootstrapping

Bootstrapping was performed to compare the differences in responsiveness estimates between the measurable data, and the results were expressed in terms of bias, standard error, and 95% confidence interval (CI). Bootstrapping is a resampling technique to draw numerous samples from the original sample with replacement. In this study, a bias-corrected bootstrap method (bias corrected accelerated, BCa) with 1000 samples and bias, standard error, and 95% confidence interval (CI) were reported. We chose 1000 iterations because this was a presumed sample size for running bootstrapping. Bootstrapping was carried out using IBM SPSS 27 (Armonk, New York).

### Statistical analysis

A direct comparison of the basic characteristic and the main outcome between different age group (≤ 65 and > 65 years old) was performed using Independent *t* test, Chi-square test or Fisher’s exact test where appropriate. Sub-group analysis was also conducted with the above tests. The primary and secondary measures were analysed based on this division. Stepwise logistic regression modelling was carried out to evaluate the possible potential factors affecting any complication being an important clinical outcome. Both crude and multivariate comparisons with confounding factors controlled were carried out (Garden type and gender). SPSS statistical software was used for analysis. Statistical significance was set as p ≤ 0.05.

## Results

233 hip fractures (68 males; 165 females) with a mean age of 73.04 ± 12.89 years who were treated by 3 cannulated screws in an inverted triangle fashion between January 2014 to June 2018 were included in the study. 165 (70.8%) were female patients and 68 (29.1%) were male patients. 195 (83.6%) patients were undisplaced with Garden type I and II, and 38 (16.3%) patients were displaced fracture with Garden type III and IV. All displaced fractures were from the “younger” hip fracture group, which were patients aged between 50–65. Regarding the premorbid condition, 147 cases were able to walk independently and 78 cases were assisted by walking aids, 8 cases were wheelchair or bedbound. The ACCI was significantly higher in the elder group at 4.96 ± 1.15 compared to the “younger” group at 3.01 ± 1.25 (p = 0.00). Premorbid status was also significant better in the “younger” group. Baseline characteristics are shown in Table 1. The mean time from admission to surgery was 33.0 h, and the average operating time was 41.1 ± 11.2 min. The mean length of stay at our acute unit was 9.1 ± 5.7 days. After acute hospital stay, 172 patients (73.8%) were discharged to our rehabilitation unit. 60 patients (25.8%) were directly discharged to home. 1 patient (0.4%) was discharged to old age home (OAH). 1 patient (0.4%) died in-patient after surgery due to medical complications.

**Table 1:** Baseline characteristics of the patients.

Characteristics	N = 233 (All patients)	N = 84 (50–65 years old, “younger” group)	Bootstrapping N = 1000			N = 149 (> 65 years old, “elder” group)	Bootstrapping N = 1000			p-value	p-value after bootstrapping N = 1000
			Bias	SE	95% CI		Bias	SE	95% CI		
Mean age (years)	73.04 ± 12.90	58.44	− 0.01	0.48	57.46–59.35	81.3 ± 7.77	− 0.03	0.63	80.10–82.35	< 0.01	< 0.01
Age (SD)		4.37	− 0.04	0.24	3.94–4.72		− 0.02	0.37	7.01–8.41		
**Gender**											
Female, n %	165, 70.8%	56, 66.7%				109, 73.2%				0.29	
Male, n %	68, 29.1%	28, 33.3%				40, 26.8%					
**Garden classification**											
I/II	195, 83.7%	46, 54.8%				149, 100%				< 0.01	
III/IV	38, 16.3%	38, 45.2%				0, 0%					
Age-adjusted Charlson Comorbidity Index (ACCI)	4.26 ± 1.49	3.01	0.00	0.14	2.73–3.28	4.96	0.00	0.09	4.79–5.14	< 0.01	< 0.01
ACCI (SD)		1.25	− 0.01	0.11	1.03–1.42	1.15	− 0.01	0.09	0.98–1.31		
**Comorbidities**											
Diabetes mellitus	49, 21.0%	15, 17.9%				34, 22.8%				0.37	
Hypertension	60, 25.8%	16, 19.0%				44, 29.5%				0.08	
Chronic lung disease	7, 3.0%	2, 2.4%				5, 3.4%				0.68	
History of stroke	14, 6.0%	2, 2.4%				12, 8.1%				0.08	
Dementia	16, 6.9%	1, 1.2%				15, 10.1%				0.01	
Renal disease	5, 2.1%	2, 2.4%				3, 2.0%				0.85	
Liver disease	13, 5.6%	8, 9.5%				5, 3.4%				0.05	
Cancer	5, 2.1%	3, 2.4%				2, 1.3%				0.26	
Peripheral vascular disease	1, 0.4%	1, 1.2%				0, 0%				0.18	
Myocardial infarction	5, 2.1%	2, 2.4%				3, 2.0%				0.85	
Congestive heart failure	5, 2.1%	0, 0%				5, 3.4%				0.09	
**Mobility status**											
Independent walker	147, 63.1%	77				72				< 0.01	
Stick walker	56, 24.0%	4				49					
Frame walker	22, 9.4%	3				20					
Wheelchair/bedbound	8, 3.4%	0				8					

Surgical outcomes showed that the complication rate of hip screw fixation for all patients was 21.5% (50 patients), with 24 from the “elderly” hip fracture group and 26 from the “younger” hip fracture group (Table 2). For the “elderly” group, the complication rate was 16.1% with 1 non-union, 5 cut-out and 18 avascular necrosis cases. For the “younger” hip fractures, complication amounted to 31.0% with 5 non-union, 1 cut-out, and 20 avascular necrosis. There were significantly more complications in the “younger” group (p = 0.008). Analysis showed that the younger group had significantly higher non-union (p = 0.01) and avascular necrosis (p = 0.02) compared to the elder group.

**Table 2:** Fracture complications and revision surgery.

	N = 233 (all patients)	N = 84 (50–65 years old, “younger” group)	N = 149 (> 65 years old, “elder” group)	p-value
**Complications**	N = 50	N = 26	N = 24	0.01
Non-union	6	5	1	
Screw cut out	6	1	5	
Avascular necrosis	38	20	18	
Infection	0	0	0	
Periprosthetic fracture	0	0	0	
**Revision surgery**	N = 25	N = 15	N = 10	0.01
Bipolar arthroplasty	12	7	5	
Total hip replacement	10	7	3	
Removal of hip screws	13	1	1	
Excisional arthroplasty	1	0	1	

The Age-adjusted Charlson Comorbidity Index (ACCI) was significantly higher in the patients with complications at 4.88 ± 1.44 compared to those without complications at 3.99 ± 1.37 (p = 0.000). More importantly, for the “younger” group with complications, the ACCI was 4.22 ± 1.34 compared to 2.62 ± 0.82 to those without complications in the same group (p = 0.000). For the “elder” group the ACCI was 5.63 ± 1.16 compared to 4.86 ± 1.11 to those without complications in the same group (p = 0.006).

Complication rates for all patients with 20 Garden Type I, 10 Garden Type II, 8 Garden Type III and 12 Garden Type IV were 14.5%, 17.5%, 40% and 66.7%, respectively. For elderly patients the complication rates for Garden I and II were 14.2% and 20.9%, respectively. There were no Garden III and IV for elderly patients as these would have undergone arthroplasty. For “younger” patients the failure rates for Garden I, II, III, and IV were 15.6%, 7.1%, 40% and 66.7%, respectively. The complications rate of the undisplaced group (Garden Type I and II) was significantly lower than the displaced group (Garden Type III and IV) (p < 0.01).

Crude analyses of stepwise logistic regression modelling were performed for all patients (Table 3). Crude analysis involving ACCI on any complication showed statistical significance in this model (p = 0.04). Positive relationships were found with ACCI = 6 (odds ratio = 4.27, p = 0.02) and CCI ≥ 7 (odds ration = 8.13, p < 0.01). The r^2^ values were comparatively better after controlled by Garden type III/IV (r^2^ = 0.29, all comparisons p < 0.01), even at ACCI = 4 (OR 6.42 (1.70, 24.25), p = 0.01). Adding gender had no effect on the model (B = − 0.03, Odds ratio = 0.97, p = 0.95). Refer to Table 3.

**Table 3:** Stepwise logistic regression models on any complication using age-adjusted Charlson Comorbidity Index (ACCI) and different confounding factors (Crude = ACCI, Model 1 = ACCI + Garden Type, Model 2 = ACCI + Garden Type + Gender). Bias, its standard error (Standard error) and 95% confidence intervals (95% CI) of B after bootstrapping for N = 1000 were performed.

Model	Factors	r^2^	SE	B	Bootstrapping N = 1000			Odds ratio (Min, Max)	p value	p value after bootstrapping N = 1000
					Bias	Standard error	95% CI			
Crude	Overall	0.08							0.04	
	Age adjusted CCI ≥ 4 (Ref: 3 or lower)									
	CCI = 4		0.57	1.04	0.09	1.03	− 0.38 to 3.09	2.82 (0.93, 8.52)	0.07	0.05
	CCI = 5		0.57	0.99	0.08	1.06	− 0.63 to 3.05	2.68 (0.88, 8.24)	0.08	0.07
	CCI = 6		0.64	1.45	0.05	1.41	0.13 to 3.07	4.27 (1.22, 14.96)	0.02	0.01
	CCI ≥ 7		0.70	2.10	0.05	1.48	− 0.28 to 4.45	8.13 (2.05, 32.26)	< 0.01	< 0.01
Model 1	Overall	0.29							< 0.01	
	Age adjusted CCI ≥ 4 (Ref: 3 or lower)									
	CCI = 4		0.68	1.86	0.31	1.99	0.15 to 20.83	6.42 (1.70, 24.25)	0.01	0.01
	CCI = 5		0.76	2.53	0.48	2.55	0.58 to 21.98	12.51 (2.82, 55.60)	< 0.01	< 0.01
	CCI = 6		0.83	2.96	0.49	2.50	0.88 to 22.31	19.22 (3.82, 96.82)	< 0.01	< 0.01
	CCI ≥ 7		0.88	3.70	0.41	2.76	0.85 to 22.60	20.57 (7.20, 68.49)	< 0.01	< 0.01
	Garden type III/IV (Ref: I/II)		0.58	2.99	0.38	2.15	1.58 to 22.12	19.87 (6.36, 62.07)	< 0.01	< 0.01
Model 2	Overall	0.29							< 0.01	
	Age adjusted CCI ≥ 4 (Ref: 3 or lower)									
	CCI = 4		0.70	1.87	0.28	1.85	0.07 to 20.80	0.22 to 20.58	0.01	< 0.01
	CCI = 5		0.77	2.54	0.41	2.00	0.73 to 21.98	0.54 to 21.84	< 0.01	< 0.01
	CCI = 6		0.84	2.97	0.35	1.99	0.83 to 23.14	0.91 to 21.67	< 0.01	< 0.01
	CCI ≥ 7		0.92	3.72	0.33	2.29	1.35 to 22.66	1.47 to 10.58	< 0.01	< 0.01
	Garden type III/IV (Ref: I/II)		0.58	2.99	0.27	1.24	1.23 to 22.68	1.68 to 5.57	< 0.01	< 0.01
	Gender: Male (Ref: Female)		0.45	− 0.03	0.04	0.50	− 1.03 to 1.13	− 0.96 to 1.14	0.95	0.95

After surgery for internal fixation, 95 patients (40.8%) walked unaided, 65 (27.9%) with a stick, 29 (12.4%) with a frame, and 32 (13.7%) were non-ambulatory. 126 patients (54.1%) required a walking aid, 46 needed assistance (19.7%), 65 (24.5%) were non-ambulant and only 95 were independent walkers. For the “younger” group, 73.8% of the patients retained function at least comparable to their premorbid. As for the “elder” group, only 51.0% retained function at least comparable to their premorbid. The summary of patient mobility pre-operative and post-operative outcomes are shown in Tables 4 and 5. Of the 50 patients that suffered from complications, 25 patients had a revision surgery within the 6 months of follow-up, with 15 in the “younger” group and 10 in the elderly group. Of the 15 “younger” patients, 7 underwent revision to total hip replacement, 7 underwent revision to bipolar hemiarthroplasty, and 1 underwent removal of hip screws. In terms of mobility after revision surgery, 6 walked unaided, 8 walked with a stick, and 1 was wheelchair bound. For the elderly group, 5 underwent revision to total hip replacement, 3 underwent hemiarthroplasty, 1 underwent removal of hip screws, and 1 underwent excisional arthroplasty. In terms of mobility after revision surgery, 1 walked unaided, 2 walked with a stick and 2 were wheelchair bound. The summary of revision surgery is shown in Table 2.

**Table 4:** Mobility changes of patients (age 65 years old or less) before and 1-year after surgery.

Mobility aid 1 year after surgery	Mobility aid before surgery in age ≤ 65 years old				
	Walks unaided (n = 77)	One stick (n = 4)	Frame (n = 3)	Wheelchair/bed bound (n = 0)	Total
Walks unaided	60 (78.0%)	0	1 (33.3%)	0	61
One stick	14 (18.2%)	1 (25%)	0	0	15
Frame	3 (3.9%)	1 (25%)	1 (33.3%)	0	5
Wheelchair/bed bound	0	2 (50%)	1 (33.3%)	0	3
Total	77 (91.7%)	4 (4.8%)	3 (3.6%)	0	84

**Table 5:** Mobility changes of patients (age > 65 years old) before and 1-year after surgery.

Mobility aid 1 year after surgery	Mobility aid before surgery in age > 65				
	Walks unaided (n = 72)	One stick (n = 49)	Frame (n = 20)	Wheelchair/bed bound (n = 8)	Total
Walks unaided	28 (38.9%)	6 (12.2%)	0	0	34
One stick	28 (38.9%)	20 (40.8%)	2 (10%)	0	50
Frame	8 (11.1%)	16 (32.7%)	12 (60%)	0	26
Wheelchair/bed bound	8 (11.1%)	7 (14.58%)	6 (30%)	8 (100%)	29
Total	72 (48.3%)	49 (32.9%)	20 (13.4%)	8 (5.4%)	149

The overall mortality rate for all patients was 20.2% (n = 47) at 1 year. Only 5 patients (6.0%) were from the “younger” fracture group, whilst the remaining 42 patients (28.2%) were from the elder group. The overall ACCI was 5.12 ± 1.61 for patients that died compared to 3.5 ± 1.71 for patients that survived (p = 0.000). The ACCI was also significantly higher for the “elder” group that died at 5.43 ± 1.40 compared to those that survived at 3.84 ± 1.91 (p = 0.000). There was no significant difference for the “younger” group (p = 0.45).

Bias and standard error of the mean and standard deviation in age and age-adjusted Charlson Comorbidity Index (ACCI) were both very low after performing bootstrapping for 1000 iterations (Table 1). The small 95% CI in mean age and ACCI and the corresponding SD in both “younger” group and “elder” group reflected these observations also (Table 1). The p-values after bootstrapping for 1000 iterations were the same as the p-values without bootstrapping (Table 1). In stepwise logistic regression models after bootstrapping, biases and standard errors were small in all models (Crude, Model 1 and Model 2) (Table 3). p-values before and after bootstrapping for 1000 iterations were similar, except for the crude model at ACCI = 4 advanced to statistical significance after bootstrapping (p values: before = 0.07, after = 0.05) (Table 3).

## Discussion

With the increasing number of hip fractures in Asia, there is a projected increase from 1.12 million in 2018 to 2.56 million in 2050^2^. China currently has a rapid increasing rate of hip fractures. In Beijing, from 2002 to 2006, there was an increase of 58% and 49% of hip fractures in women and men, respectively^23^. Being a debilitating disease and causing a high socioeconomic cost, further epidemiological and updates in optimal clinical management are warranted. The current standard and guidelines in operating a hip fracture is within 48 h of admission, which has been proven to decrease mortality and complications^24^. A previous systematic review of 52 studies involving 291,413 patients, also show that an early surgery within 48 h also reduces hospital stay^25^. Our patients were operated at a mean time of 33 h, which was within this recommendation. Understandably, the ACCI and premorbid walking status were better in the “younger” group compared to the “elder” group. The overall complication rate was 21.5% for our cases. In fact, numerous studies have shown that joint salvage procedures for undisplaced neck of femur fractures have high risk of complications, revision, non-union and poor function with failure rates reaching 40% or more^26^. Interestingly, a recent systematic review of 1971 patients with 16 published studies also recommended arthroplasty surgery as a reasonable option in elderly patients with an undisplaced neck of femur fracture due to the high risk of complications^27^. This further reinforces the controversy as to whether we should still use internal fixation for these patients. Our research question was to identify the risk factors that have high rates of complications in hip screw fixation, to further improve current clinical management.

The overall surgical outcomes in our study showed that the complication rate of hip Screw fixation for all patients was 21.5%. More importantly, the ACCI was significantly higher in patients with complications compared to those without complications in both “younger” and “elder” groups. Previous studies have shown that initial fracture displacement and fracture reduction are important parameters to determine the risk of hip screw failure^28^. In fact, even for patients that have initial valgus and posterior tilt > 15°, the risk of fixation failure and avascular necrosis is significantly higher^29^. Meta-analyses also show that the Garden Classification and retained implants are important factors of avascular necrosis of the femoral head^30^. Regarding comorbidities, it has been found that chronic kidney disease^31^, respiratory disease^13^ and low serum albumin levels^32^ are also risk factors of failure. However, we present the first study to identify ACCI as an indicator for high risk of surgical complications after hip screw fixation in neck of femur fractures. It has generally been shown that Charlson Comorbidity Index (CCI) is correlated with increased mortality in fragility fracture patients^33^. Patients with a higher CCI have significantly longer time-to-surgery, length of hospital stay, transfusion, and osteoporosis in elderly hip fracture patients^34^. Furthermore, recent evidence shows that the CCI is a strong predictor of complication rates for patients undergoing revision total hip arthroplasty^35^. A previous nationwide Danish study of 36,984 patients also showed that a high CCI score were predictive of failure in primary total hip replacement, irrespective of the period of follow-up^36^. Our results concur similarly with these findings. It is well known that the ACCI includes age, stroke, diabetes, liver and renal diseases, which are strongly associated with osteoporosis and bone resorption^36^. The affected bone metabolism would understandably lead to poor bone quality. Furthermore, vascular disease from comorbidities can also affect blood supply and healing. Given the current results, an ACCI ≥ 6 in a neck of femur fracture elderly patient, or a ACCI ≥ 4 in a displaced neck of femur fracture patient, mainly in those that are 50–65 years old, would have high risk of complications or failure in 1 year.

Our current results show that in the Chinese population, displaced neck of femur fractures fixed with cannulated hip screws have high complication rates with 40% with Garden Type III and 66.7% with Garden Type IV. These displaced neck of femur fractures would be present in our “younger” patient cases as joint salvage procedure is preferred^9^ and a typical arthroplasty would not last for over 20 years^8,37,38^. Furthermore, younger patients typically have better bone quality^39^. However, failure is also recognizably high in these patients as the blood supply to the femoral head is disrupted with non-union and avascular necrosis being the most common complications^27^. The dilemma in the best management due to varying failure rates^13^ has led to controversy towards displaced neck of femur fractures in younger hip fractures patients at 50–65 years of age^8^. As with existing literature, a Garden Type III or IV has high risk of failure. The matter of how to improve the outcomes for these patients is still a major challenge to Orthopaedic Surgeons. However, the authors would recommend that in patients with a displaced neck of femur fracture and an ACCI ≥ 4, a direct arthroplasty surgery should be considered.

Conversion to total hip arthroplasty is a well-accepted and effective method to salvage failed fixation of hip fractures. Numerous studies have shown positive results in pain relief and function after revision^40,41^. A previous study by Archibeck et al. showed that total hip arthroplasties in a series of 102 failed internal fixation of proximal femoral fractures were clinically successful with a mean Harris Hip Score of 81.8 at a minimum of 2-year follow-up. However, there were high complication rates (11.8%) with 5 dislocations, 4 periprosthetic fractures, 2 hematomas and 1 infection^42^. Furthermore, another study by Moon et al. had a series of 96 total hip arthroplasties following failed fixation of 59 femoral neck and 37 intertrochanteric fractures. It was shown that the Harris Hip Score was significantly improved as well^43^. It is well known that a conversion total hip arthroplasty after an initial failed fixation has more complications than a primary total hip arthroplasty^44^ due to the increased difficulty of surgery from scar tissue and bone stock. In fact, a recent study by Douglas et al. showed that conversion has more than twice the complication rate compared to a primary total hip replacement^45^. These include periprosthetic joint infections (7.7% vs. 1.4%), hip dislocations (4.5% vs. 2.0%), blood transfusion (2.0% vs. 1.0%), mechanical complications (5.5% vs. 1.0%), and revision surgeries (4.0% vs 1.5%)^45^. As a result, it would be important to consider whether a direct arthroplasty surgery should have been performed at the initial stage for the correct candidate, as this would potentially decrease complications and healthcare costs.

In terms of function, for the “younger” group, 73.8% of the patients retained function at least comparable to their premorbid. As for the “elder” group, only 51.0% retained function at least comparable to their premorbid. It is well known that postoperative mobility compared with premorbid deteriorates with time for a hip fracture patient. In fact, a review study showed that only 40–60% of patients recover their pre-fracture level of mobility and ability to perform instrumental activities of daily living. 40–70% of patients regain ability of basic levels of activity^46^. This concurs with our current results. However, at 1-year and 5-years after a hip fracture, there is increased dependency for mobility and decreased physical quality of life^47^. The function after a conversion total hip arthroplasty is also worse than a primary total hip arthroplasty by 3 years^45^. This further shows the importance in identifying the neck of femur fractures patients that have high risk of failure to consider for a direct arthroplasty surgery.

Overall mortality of our cases was 20.2% at 1 year. The general mortality of hip fracture patients varies from 14–58%^48^. Several risk factors of death have been identified including dementia, diabetes mellitus, and pneumonia in a study by Muraki et al.^49^. Furthermore, a recent study with a large cohort study of 36,082 hip fracture patients found that patients of Malay ethnicity, older age, male sex, pre-fracture comorbidity, and trochanteric fractures have increased risk of death^50^. Our results also concur that there was a significantly higher ACCI in those that died compared to patients that survived. The overall ACCI was 5.12 ± 1.61 for patients that died compared to 3.5 ± 1.71 for patients that survived (p = 0.000). The ACCI was also significantly higher for the “elder” group that died compared to those that survived. Understandably the mortality rate in the “younger” group was much lower than the elder group leading to no significant differences. Similarly other studies have also found that CCI correlates with mortality in hip fracture patients^33^.

The sample size in this study was 233, with 84 patients in the “younger” group and 149 patients in the “elder” group, from a single institute. The statistical power of this study was projected to draw accurate conclusions after obtaining promising results from bootstrapping. Results from bootstrapping for 1000 iterations showed small 95% confidence intervals in age and age-adjusted CCI (i.e., two important baseline characteristics) and regression coefficients (i.e., B). Similar p-values before and after 1000 iteration further support the adequate statistical power in this study. The only change in the results from insignificant to significant p-value after bootstrapping at ACCI = 4 in the crude model, but would not affect the final outcomes in this study because it requires controlling of confounding factors. Results from model 1 and model 2 reflect the observations.

The limitations of this study are that different surgeons operated for the cases, which may have varying results. Our follow-up was only up to 1 year and did not provide the long-term results of the hip fracture patients. The included patients were neck of femur fractures only, and we studied on fracture-related complications but not perioperative complications as current literature has shown that this risk is low with hip screws fixation^6^. Furthermore, studies have shown that the modified Elixhauser’s Comorbidity Measure achieves a very good prediction of complications following hip fractures and have been found to be superior to the Charlson Comorbidity Index^51^, although some studies have shown similar accuracies in terms of mortality risk^52^. We adopted ACCI as it has been published in numerous studies that the score correlates with mortality, complications, and readmission rates after treatment of hip fractures^22,33,53–55^, and has been used and published in our locality^56^. The strength of this study is that it is the first to correlate ACCI with high risk of complications for hip screw fixation in neck of femur fracture. Based on these results, we are able to recognize the risk factors of high failure rate and the authors would recommend a direct arthroplasty for patients with a displaced neck of femur fracture and an ACCI ≥ 4, as well as patients with an ACCI ≥ 6. Given the rising number of hip fractures, optimal management of these patients are warranted to decrease complications and revision surgery. The incorporation of ACCI in our clinical pathways may prove to be useful.
